# Supplementary material for: Neural deficits in a mouse model of PACS1 syndrome are corrected with PACS1- or HDAC6-targeting therapy
Source: Nat Commun. 2023 Oct 17;14:6547. doi: 10.1038/s41467-023-42176-8 (PMC10582149; doi:10.1038/s41467-023-42176-8)
Supplement: Supplementary file 1 — Suppementary Information [file 41467_2023_42176_MOESM1_ESM.pdf]

Table S1

PCR primers used to genotype the mouse lines described in this study. Gene schematics depicting each primer binding site and representative PCR gels are shown in Figures S1c, S3a, and S7a.

Genotyping PCR primers	
**Primer Name**	**Sequence (5' - 3')**
e4F	GGTACAAGAACCGAACTATCT
eD4F	GGTACAAGAACCGAACTTGG
i4R	CTGGTCTACAAAGTGAGTTCC
NeoF	GCTAACCATGTTCATGCCTTC
NeoR	CGTTGGCTACCCGTGATATT
R26F	GCCTCCTGGCTTCTGAGGACCG
R26R	TCTGTGGGAAGTCTTGTCCCTCC
SAR	CCTGGACTACTGCGCCCTACAGA
GFPF	AGGACGACGGCAACTACAAG
GFPR	GTCCATGCCGAGAGTGATCC
i3F	TCGTCATAATCCTGGTGACTGT
e4R	CCTGCAGGCTGGAGAAACA

Table S2

qRT-PCR primers used to measure the indicated mRNAs from mouse cortex as shown in Figure 6c.

**RT-qPCR primers**	
**primer name**	**sequence (5' - 3')**
Gapdh F	CATGGCCTTCCGTGTTCCTA
Gapdh R	GCCTGCTTCACCACCTTCTT
Pacs1 F	AGACACCACCAGTCCTATGG
Pacs1 R	GCAGATCAGCTTTGCTGGAC
Hdac6 F	CACCGCATTCAGAGGGTTCT
Hdac6 R	CCTTAAGGTGGGGCCAGAAG
Pacs2 F	GAAAACCCAAGAAGCAGCGG
Pacs2 R	GCTCCGAATCCAAGACCTCC
Wdr37 F	ACCACATCCAGGGCCATCTGT
Wdr37 R	CTATGCTCCACAACAAAGCCGT

Supplemental Figure Legends

Figure S1: **(a)** (Left) $PACS1^{R203W}$ patient (650) and healthy parent (651) fibroblasts were fixed and stained for GM130 or Giantin (red, only GM130 staining is shown) and nuclei (DAPI). (Right) Quantification of Golgi fragmentation and dispersal, as described in Fig. 1. Data are mean ± SEM (2-tailed t-test), n = 51 (650, Giantin), 51 (651, Giantin), 57 (650, GM130), or 60 (651 GM130) cells/group from three independent experiments. Scale bar, 20 µm. **(b)** Patient (159) and parent (160) cells were exposed to 5 µM nocodazole for 10 hr to depolymerize MTs. After a 3-min washout, cells were fixed and stained for EB1 (green), Pericentrin (red), and nuclei (DAPI). Arrows, asters. Data are mean ± SEM (2-tailed t-test), n = 23 (160) or 25 (159) cells /group from three independent experiments. Scale bar, 20 µm. **(c)** (Top) Schematic of *Pacs1* exon 4 harboring the 4 bp deletion in the $Pacs1^{HET}$ mice. PCR primer binding sites are indicated with arrows and their sequences are presented in Table S1. (Bottom) PCR genotyping gel showing the PCR primers used and the resultant PCR products that identify $Pacs1^{WT}$, $Pacs1^{HET}$, and $Pacs1^{KO}$ mice. **(d)** Western blot of total brain lysate prepared from *WT* and $Pacs1^{KO}$ mice. Numerical values depict the normalized signal intensity of PACS2, HDAC6, and WDR37 bands. **(e)** U2OS cells were fixed and stained for α-tubulin (red) and endogenous PACS1 (green). Arrowheads, segments of MTs that colocalize with endogenous PACS1. Scale bar, 10 µm. Source data are presented at the end of this file.

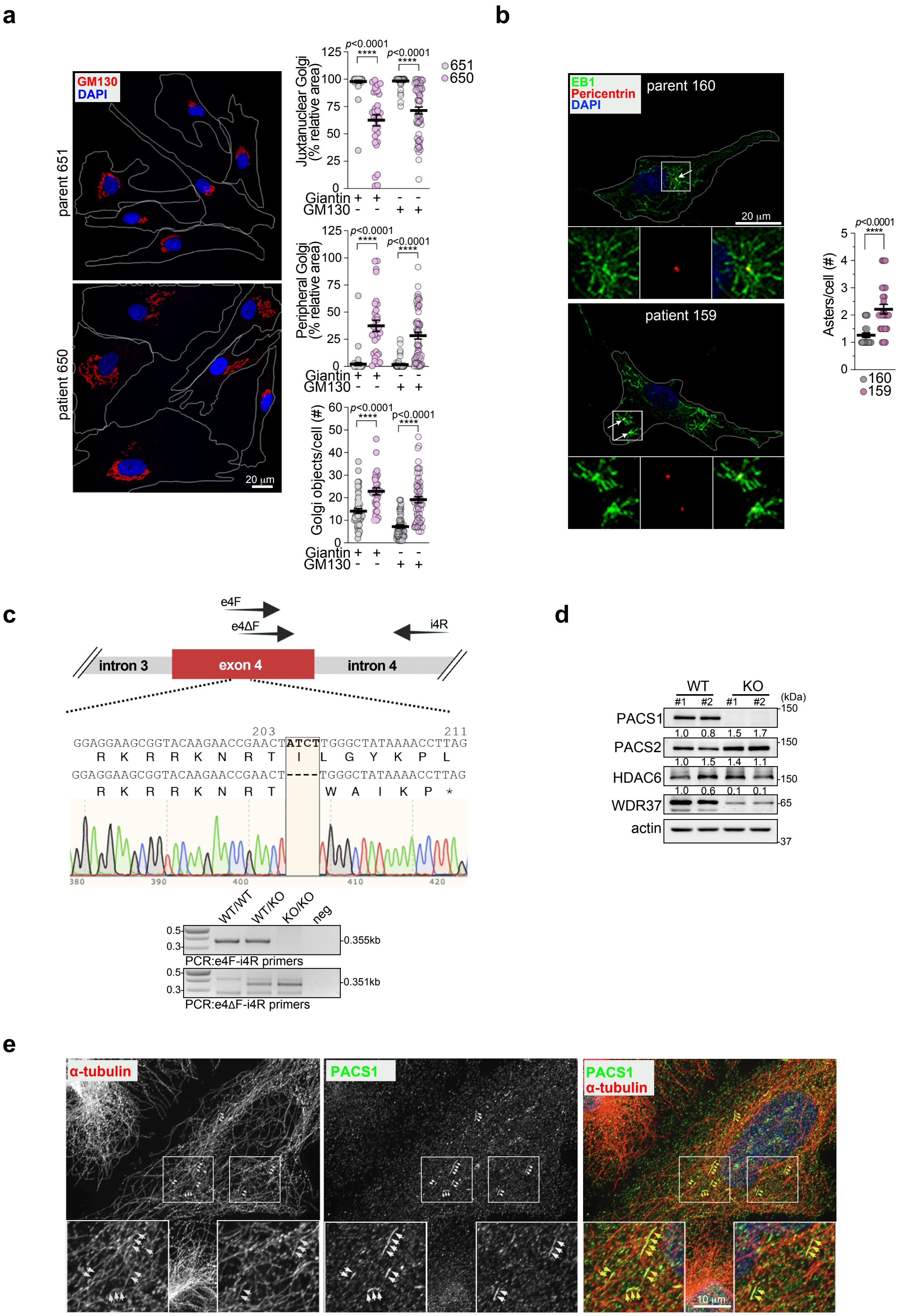
a
GM130
DAPI
parent 651
patient 650
20 µm
Juxtanuclear Golgi (% relative area)
Peripheral Golgi (% relative area)
Golgi objects/cell (#)
p<0.0001
****
651
650
Giantin + + - -
GM130 - - + +
b
EB1
Pericentrin
DAPI
parent 160
patient 159
20 µm
Asters/cell (#)
160
159
c
e4F
e4ΔF
i4R
intron 3
exon 4
intron 4
203
211
GGAGGAAGCGGTACAAGAACCGAACTATCTTGGGCTATAAAACCTTAG
R K R R K N R T I L G Y K P L
GGAGGAAGCGGTACAAGAACCGAACT----TGGGCTATAAAACCTTAG
R K R R K N R T W A I K P *
WT/WT
WT/KO
KO/KO
neg
0.355kb
PCR:e4F-i4R primers
0.351kb
PCR:e4ΔF-i4R primers
d
WT
KO
#1 #2 #1 #2
(kDa)
PACS1
1.0 0.8 1.5 1.7
PACS2
1.0 1.5 1.4 1.1
HDAC6
1.0 0.6 0.1 0.1
WDR37
actin
e
α-tubulin
PACS1
PACS1
α-tubulin
10 µm

Fig. S1

Figure S2: (a) Patient (159) and parent (160) cells were treated with vehicle alone (DMSO) or with HDAC6 inhibitors SW-100 (5 µM, 16h), or ACY1215 (2.5 µM, 16 h). Cells were fixed and stained for Giantin (red) and nuclei (DAPI). (Bottom) Quantification of Golgi fragmentation and dispersal, as described in Fig. 1a. Data are mean ± SEM (2-way ANOVA followed by Tukey *post hoc* test), n = 49 (160/veh), 58 (159/veh), 67 (160/SW-100), 73 (159/SW-100), and 34 (160/veh), 34 (159/veh), 36 (160/ACY-1215), 39 (159/ACY-1215) cells/condition from three independent experiments. Scale bar, 20 µm. **(b)** 159 and 160 cells, nucleofected with a non-specific (NS), PACS1- or HDAC6-targeting siRNA, were harvested for Western blot (left) or processed for confocal imaging (right) and analyzed as in (a), after staining for Giantin (red) and nuclei (DAPI). Data are mean ± SEM (2-way ANOVA followed by Tukey *post hoc* test), n = 96 (160/ns), 76 (160/siPACS1), 86 (160/siHDAC6), 44 (159/ns), 90 (159/siPACS1) and 96 (159/siHDAC6) cells/group from three independent experiments. Scale bar, 20 µm. **(c)** Endogenous HDAC6 was immunoprecipitated from 160 cells and 159 cells, and bound PACS1 was detected by Western blot. IgG was used as the negative control. Data are mean ± SEM (2-tailed t-test), n = 3 independent experiments, normalized individually to minimize inter-experimental variability. **(d)** HCT116 cells co-expressing HDAC6-V5 and the FLAG-tagged $PACS1^{1-266}$ (construct D in Figure 2f) or R203W-$PACS1^{1-266}$ were harvested and FLAG-tagged proteins captured with M2 agarose. Co-precipitating HDAC6-V5 was detected by Western blot. Data are mean ± SEM (2-tailed t-test), n = 3 independent experiments, normalized individually to minimize inter-experimental variability. Source data are presented at the end of this file.

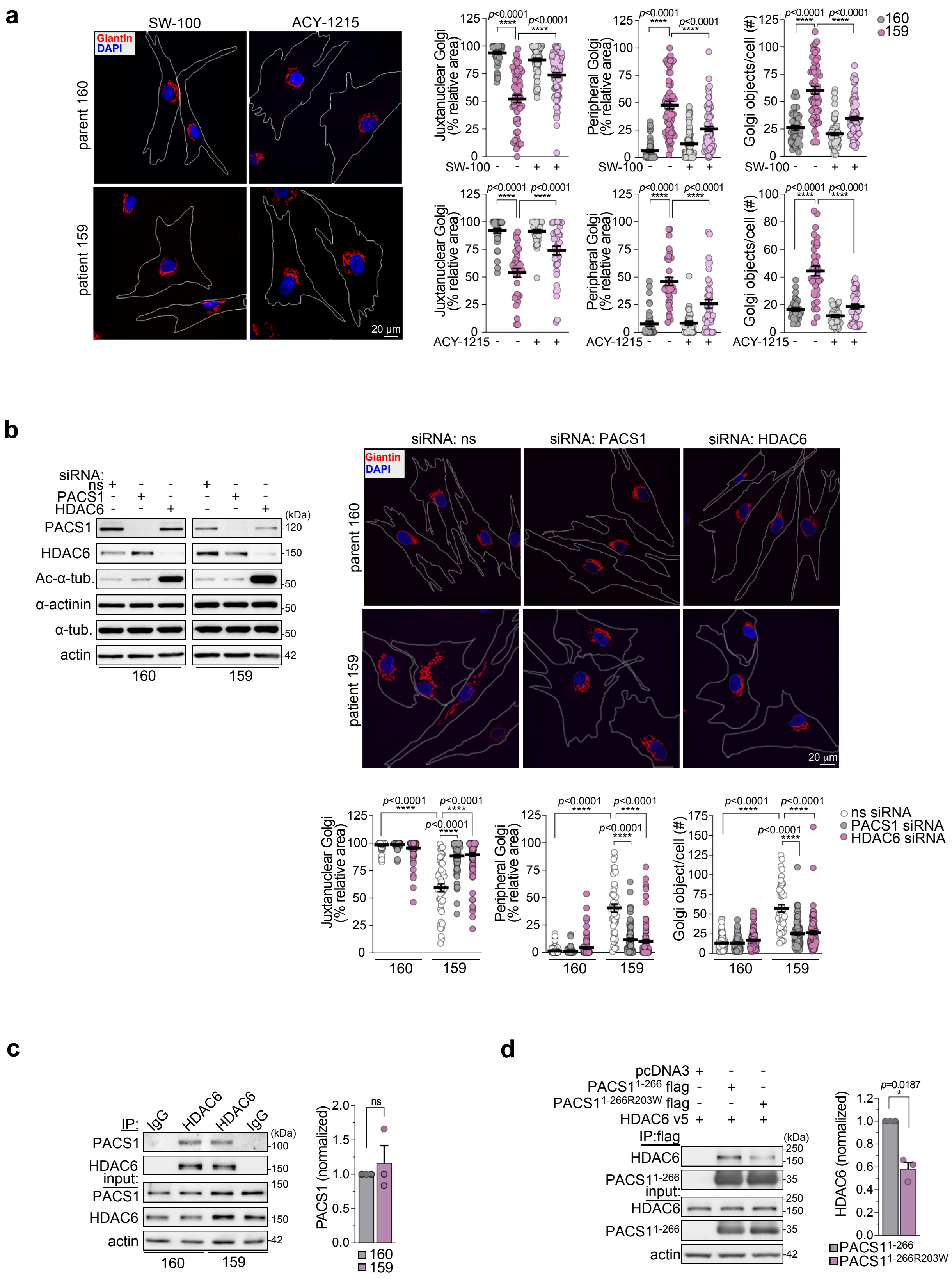
a
SW-100
ACY-1215
Giantin
DAPI
parent 160
patient 159
20 μm
Juxtanuclear Golgi (% relative area)
Peripheral Golgi (% relative area)
Golgi objects/cell (#)
p<0.0001
****
SW-100
ACY-1215
160
159
b
siRNA:
ns
PACS1
HDAC6
(kDa)
PACS1
HDAC6
Ac-α-tub.
α-actinin
α-tub.
actin
120
150
50
42
160
159
siRNA: ns
siRNA: PACS1
siRNA: HDAC6
Giantin
DAPI
parent 160
patient 159
20 μm
Juxtanuclear Golgi (% relative area)
Peripheral Golgi (% relative area)
Golgi object/cell (#)
ns siRNA
PACS1 siRNA
HDAC6 siRNA
c
IP:
IgG
HDAC6
HDAC6
IgG
(kDa)
PACS1
HDAC6
input:
PACS1
HDAC6
actin
100
150
42
160
159
PACS1 (normalized)
ns
d
pcDNA3
PACS1¹⁻²⁶⁶ flag
PACS1¹⁻²⁶⁶ᴿ²⁰³ᵂ flag
HDAC6 v5
IP:flag
(kDa)
HDAC6
PACS1¹⁻²⁶⁶
input:
HDAC6
PACS1¹⁻²⁶⁶
actin
250
150
35
42
HDAC6 (normalized)
p=0.0187
*
PACS1¹⁻²⁶⁶
PACS1¹⁻²⁶⁶ᴿ²⁰³ᵂ

Fig. S2

Figure S3: **(a)** (Top) CRISPR/Cas9 methods were used to insert floxed cassettes designed to express HA-tagged PACS1 or $PACS1^{R203W}$ under control of the CAG promoter into the Rosa26 safe harbor locus. PCR primer binding sites are indicated with arrows and their sequences are presented in Table S1. The IRES-dependent expression of the GFP sequence downstream of the PACS1 or $PACS1^{R203W}$ cDNAs was too low to be detected and was used only for PCR genotyping. (Bottom) PCR genotyping gel depicting the PCR primers used and the resultant PCR products that identify WT, $R26^{P1}$, and $R26^{P1R203W}$ lines **(b)** IHC of coronal brain sections prepared from P11 $Emx1^{Cre};R26^{+}$, $Emx1^{Cre};R26^{P1}$ or $Emx1^{Cre};R26^{P1R203W}$ mice were stained for CTIP2 (green), SATB2 (red) and nuclei (DAPI). Scale bar, 250 µm. **(c)** Dissociated hippocampal neurons isolated from $Emx1^{Cre};R26^{P1}$ or $Emx1^{Cre};R26^{P1R203W}$ mice at P0 were processed for confocal imaging at DIV5. High-resolution tiled images of entire cultured hippocampal neurons were captured using a confocal microscope (see Methods and Figure 3d). Shown is a large-format 300 DPI maximum intensity projection (MIP) TIFF image of the entire neuron depicting β3-tubulin (pseudocolored white), Giantin (pseudocolored green), HA-tagged PACS1 or $PACS1^{R203W}$ (pseudocolored red), and DAPI (blue). Arrowheads indicate colocalization of Giantin and $PACS1^{R203W}$ in varicosities along the developing neurites. Scale bar, 50 µm. The images were processed with Imaris and are presented in Figure 3d and Movie S1. Source data are presented at the end of this file.

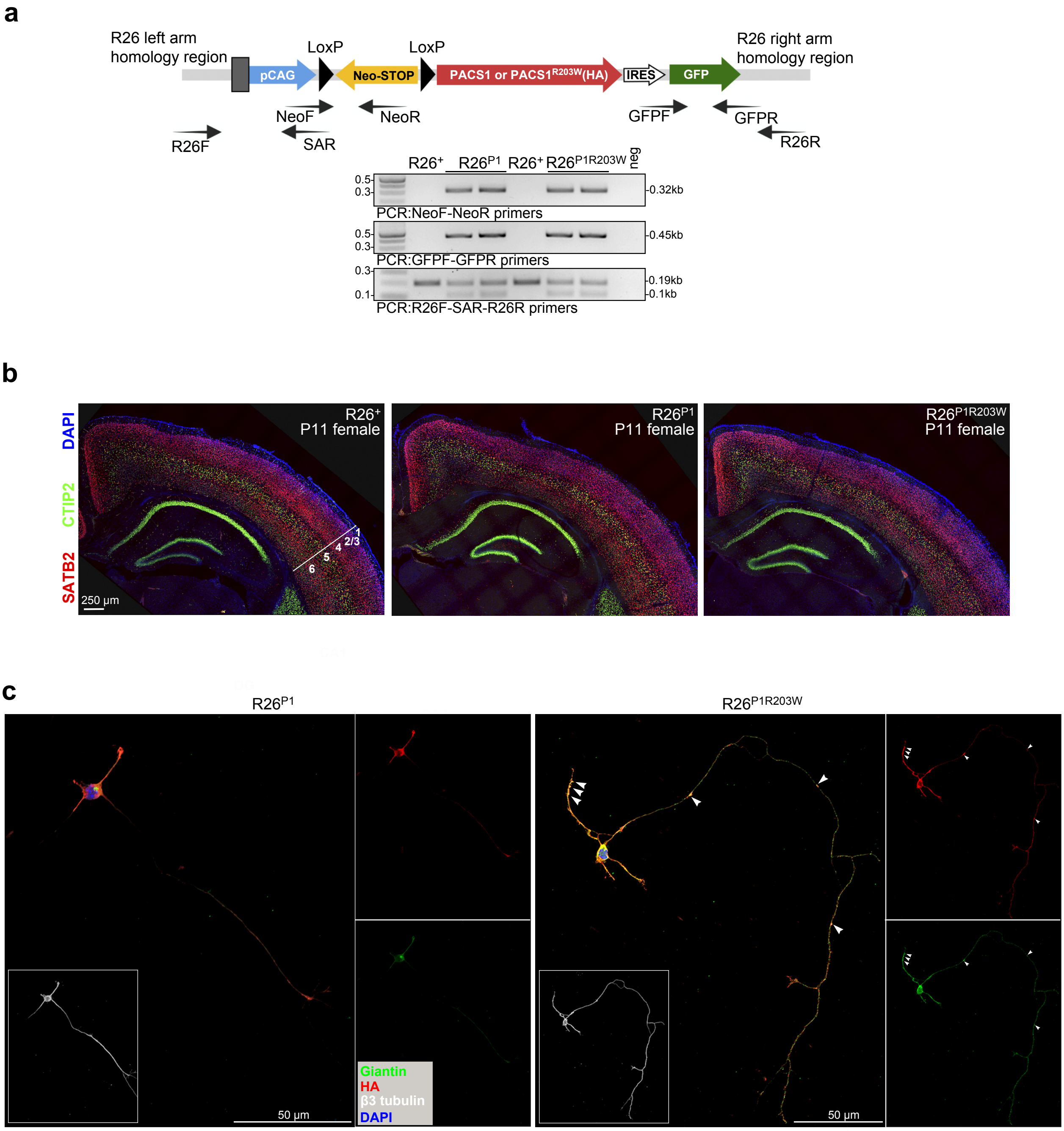
a
R26 left arm
homology region
LoxP
LoxP
pCAG
Neo-STOP
PACS1 or PACS1R203W(HA)
IRES
GFP
R26 right arm
homology region
NeoF
NeoR
SAR
R26F
GFPF
GFPR
R26R
R26+
R26P1
R26+
R26P1R203W
neg
0.5
0.3
0.32kb
PCR:NeoF-NeoR primers
0.5
0.3
0.45kb
PCR:GFPF-GFPR primers
0.3
0.1
0.19kb
0.1kb
PCR:R26F-SAR-R26R primers
b
DAPI
CTIP2
SATB2
R26+
P11 female
R26P1
P11 female
R26P1R203W
P11 female
1
2/3
4
5
6
250 μm
c
R26P1
R26P1R203W
Giantin
HA
β3 tubulin
DAPI
50 μm
50 μm

Fig. S3

Figure S4: (a) WT C57BL/6 pups were injected (ICV) at P1 with a single dose of H6ASO or control nASO (40 µg). After 4 weeks, brains were harvested and the *Hdac6* mRNA and protein levels were analyzed by qRT-PCR or Western blot, respectively. Data are mean ± SEM (2-tailed t-test), n = 4 animals/condition. **(b)** Patient (159) and parent (160) NPCs were fixed and stained for Nestin, PAX6, SOX2, and DAPI (blue). Scale bar, 20 µm. Source data are presented at the end of this file.

a

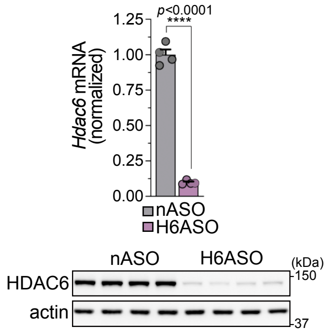
p<0.0001
****
1.25
1.00
0.75
0.50
0.25
0.00
Hdac6 mRNA (normalized)
nASO
H6ASO
nASO
H6ASO
(kDa)
HDAC6
150
actin
37

b

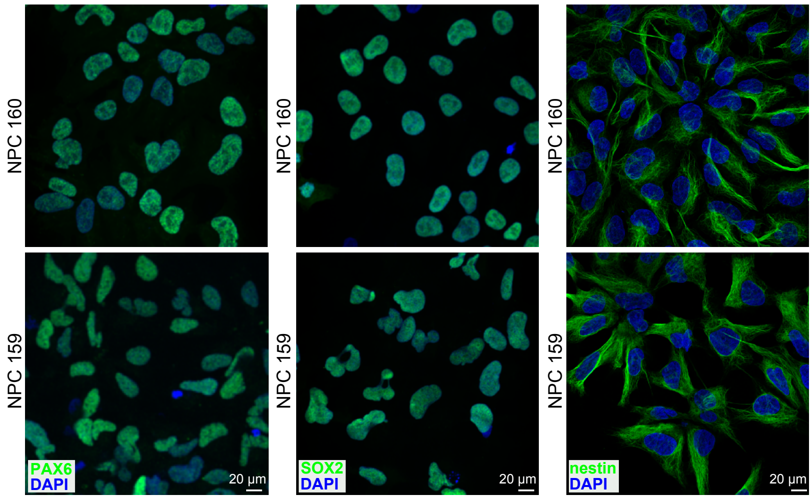
NPC 160
NPC 160
NPC 160
NPC 159
NPC 159
NPC 159
PAX6
DAPI
20 µm
SOX2
DAPI
20 µm
nestin
DAPI
20 µm

Fig. S4

Figure S5: (a) Western blot of synaptosomal fractions isolated from WT C57BL/6 forebrains. **(b)** (Left) Representative whole-cell voltage-clamp recordings of mIPSCs from L2/3 pyramidal neurons in acute brain slices of juvenile $Emx1^{Cre};R26^{P1}$ or $Emx1^{Cre};R26^{P1R203W}$ mice injected (ICV) at P1 with 40 μg nASO or H6ASO. (Middle) Summary data for mIPSC amplitude (top) and frequency (bottom). Data are mean ± SEM (2-way ANOVA followed by Tukey *post hoc* test). (Right) Cumulative probability distributions of mIPSC amplitudes (top) and frequencies (bottom). n = 17 neurons/5 mice ($R26^{P1}$/nASO), 16 neurons/5 mice ($R26^{P1R203W}$/nASO). 12 neurons/3 mice ($R26^{P1}$/H6ASO), 13 neurons/3 mice ($R26^{P1R203W}$/H6ASO). Source data are presented at the end of this file.

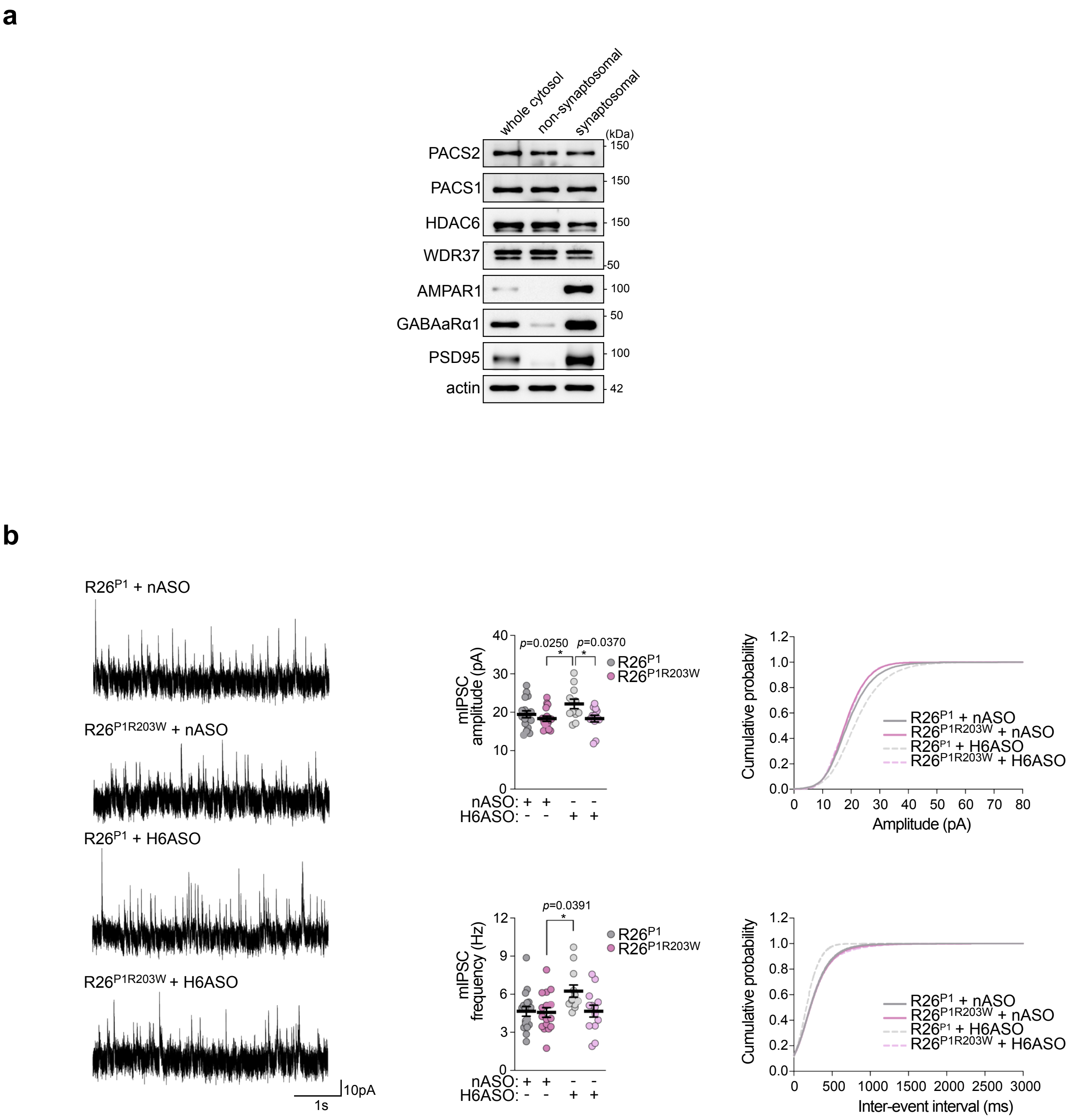
a
whole cytosol
non-synaptosomal
synaptosomal
(kDa)
PACS2
150
PACS1
150
HDAC6
150
WDR37
50
AMPAR1
100
GABAaRα1
50
PSD95
100
actin
42
b
R26P1 + nASO
R26P1R203W + nASO
R26P1 + H6ASO
R26P1R203W + H6ASO
10pA
1s
mIPSC amplitude (pA)
p=0.0250
p=0.0370
*
*
R26P1
R26P1R203W
nASO: + + - -
H6ASO: - - + +
Cumulative probability
R26P1 + nASO
R26P1R203W + nASO
R26P1 + H6ASO
R26P1R203W + H6ASO
Amplitude (pA)
mIPSC frequency (Hz)
p=0.0391
*
R26P1
R26P1R203W
nASO: + + - -
H6ASO: - - + +
Cumulative probability
R26P1 + nASO
R26P1R203W + nASO
R26P1 + H6ASO
R26P1R203W + H6ASO
Inter-event interval (ms)

Fig. S5

Figure S6: **(a)** Body mass of 6 wk old *WT*, *Pacs1*^{HET}, and *Pacs1*^{KO} male and female littermates. Data are mean ± SEM, (1-way ANOVA followed by Tukey *post hoc* test). n = 4 (WT/WT), 9 (WT/KO) and 12 (KO/KO) male mice plus 6 (WT/WT), 5 (WT/KO) and 7 (KO/KO) female mice. **(b)** Western blot of Ac-cortactin, total cortactin, and PACS1 in brain extracts prepared from adult *WT* and *Pacs1*^{KO} mice. **(c)** (Left) Representative whole-cell voltage-clamp recordings of mEPSCs from L2/3 pyramidal neurons in acute brain slices of juvenile *WT* and *Pacs1*^{KO} mice. (Middle) AMPAR mEPSC amplitude (top) and frequency (bottom) of *WT* and *Pacs1*^{KO} mice. Data are mean ± SEM (2-tailed t-test). (Right) Cumulative probability distributions of AMPAR mEPSC amplitudes (top) and frequencies (bottom). n = 12 neurons/4 mice (WT) and 10 neurons/3 mice (KO). **(d)** Representative whole-cell voltage-clamp recordings of mIPSCs from L2/3 pyramidal neurons in acute brain slices of juvenile *WT* and *Pacs1*^{KO} mice. (Middle) mIPSC amplitude (top) and frequency (bottom) of juvenile *WT* and *Pacs1*^{KO} mice. Data are mean ± SEM (2-tailed t-test). (Right) Cumulative probability distributions of mIPSC amplitudes (top) and frequencies (bottom) n = 15 neurons/4 mice (WT) and 10 neurons/3 mice (KO). Source data are presented at the end of this file.

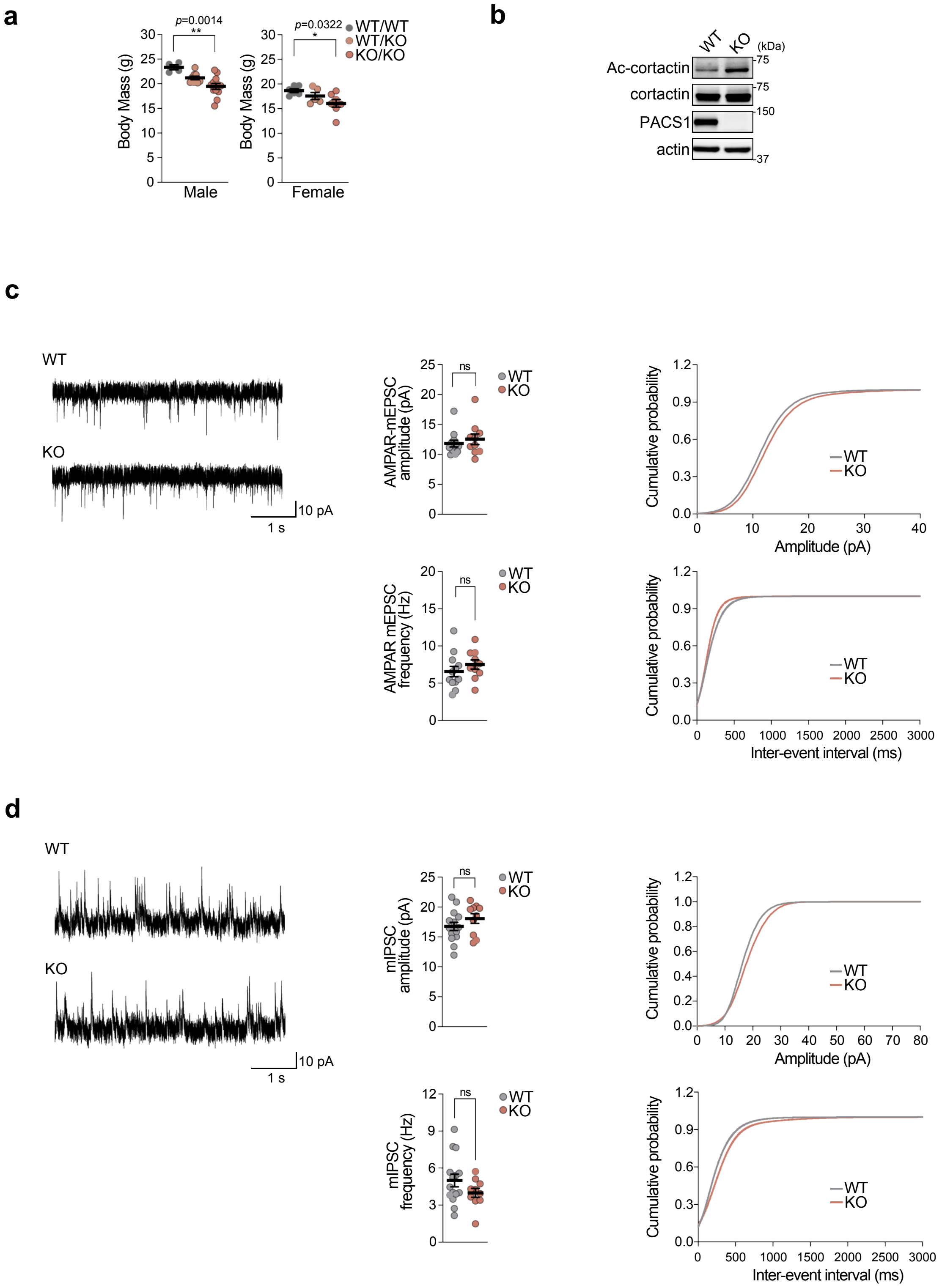

a
Body Mass (g)
Male
Female
p=0.0014
**
p=0.0322
*
WT/WT
WT/KO
KO/KO
b
WT
KO
(kDa)
Ac-cortactin
cortactin
PACS1
actin
75
75
150
37
c
WT
KO
10 pA
1 s
AMPAR-mEPSC amplitude (pA)
AMPAR mEPSC frequency (Hz)
ns
Cumulative probability
Amplitude (pA)
Inter-event interval (ms)
d
mIPSC amplitude (pA)
mIPSC frequency (Hz)

Fig. S6

Figure S7: (a) (Top) CRISPR-Cas9 methods were used to insert Megamer cassettes designed to conditionally express $PACS1^{R201W}$ or PACS1. PCR primer binding sites are indicated with arrows and their sequences are listed in Table S1. (Bottom left) PCR genotyping gel depicting the PCR primers used and the resultant PCR products that identify $Pacs1^{M/+}$ and $Pacs1^{R201W/+}$ lines. (Bottom right) Western blot showing the *Emx1*^{Cre} induction of the floxed *Pacs1* and *Pacs1*^{R201W} alleles. **(b)** Representative whole-cell voltage-clamp recordings of mIPSCs from L2/3 pyramidal neurons in acute brain slices of juvenile *Emx1*^{Cre}*;Pacs1*$^{M/+}$ and *Emx1*^{Cre}*;Pacs1*$^{R201W/+}$ mice injected(ICV) at P1 with 40 µg nASO or P1ASO. (Middle) mIPSC amplitude (top) and frequency (bottom). Data are mean ± SEM (2-way ANOVA followed by Tukey *post hoc* test). (Right) Cumulative probability distributions of mIPSC amplitudes (top) and frequencies (bottom). n = 14 neurons/6 mice ($Pacs1^{M/+}$/nASO), 14 neurons/4 mice ($Pacs1^{R201W/+}$/nASO), 13 neurons/5 mice ($Pacs1^{M/+}$/P1ASO), 14 neurons/4 mice ($Pacs1^{R201W/+}$/P1ASO). **(c)** P1 *WT* C57BL/6 pups were injected (ICV) at P1 with a single dose of P1ASO or nASO (40 µg). After 4 weeks, brains were harvested, and *Pacs1* mRNA and protein levels were analyzed by qRT-PCR and Western blot. Data are mean ± SEM (2-tailed t-test), n = 4 (nASO) or 3 (P1ASO) mice/condition. **(d)** Quantification of western blot signals for PACS1, PACS2, HDAC6, and WDR37 in the cortical lysates shown in Figure 6b. Data are mean ± SEM (2-way ANOVA followed by LSD *post hoc* test), n = 3 independent experiments, normalized individually to minimize inter-experimental variability. Source data are presented at the end of this file.

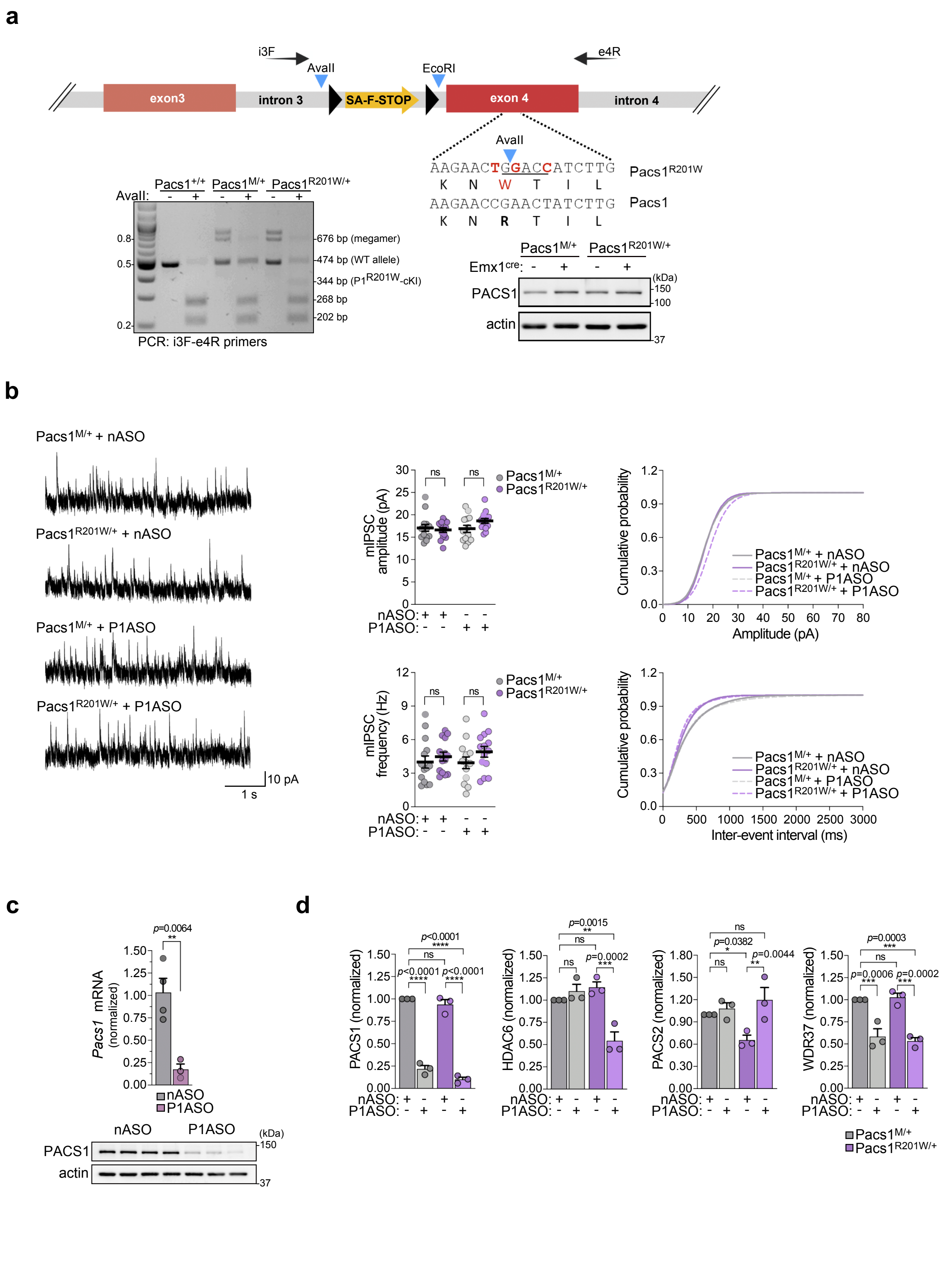
a
i3F
AvaII
EcoRI
e4R
exon3
intron 3
SA-F-STOP
exon 4
intron 4
AvaII
AAGAACTGGACCATCTTG
K N W T I L
Pacs1R201W
AAGAACCGAACTATCTTG
K N R T I L
Pacs1
Pacs1+/+ Pacs1M/+ Pacs1R201W/+
AvaII:
676 bp (megamer)
474 bp (WT allele)
344 bp (P1R201W-cKI)
268 bp
202 bp
PCR: i3F-e4R primers
Pacs1M/+ Pacs1R201W/+
Emx1cre:
PACS1
actin
(kDa)
150
100
37
b
Pacs1M/+ + nASO
Pacs1R201W/+ + nASO
Pacs1M/+ + P1ASO
Pacs1R201W/+ + P1ASO
10 pA
1 s
mIPSC amplitude (pA)
mIPSC frequency (Hz)
nASO:
P1ASO:
ns
Cumulative probability
Amplitude (pA)
Inter-event interval (ms)
c
Pacs1 mRNA (normalized)
p=0.0064
nASO
P1ASO
PACS1
actin
d
PACS1 (normalized)
HDAC6 (normalized)
PACS2 (normalized)
WDR37 (normalized)
p<0.0001
p=0.0015
p=0.0002
p=0.0382
p=0.0044
p=0.0003
p=0.0006
Pacs1M/+
Pacs1R201W/+

Fig. S7

PCR: e4F-i4R

PCR: e4ΔF-i4R

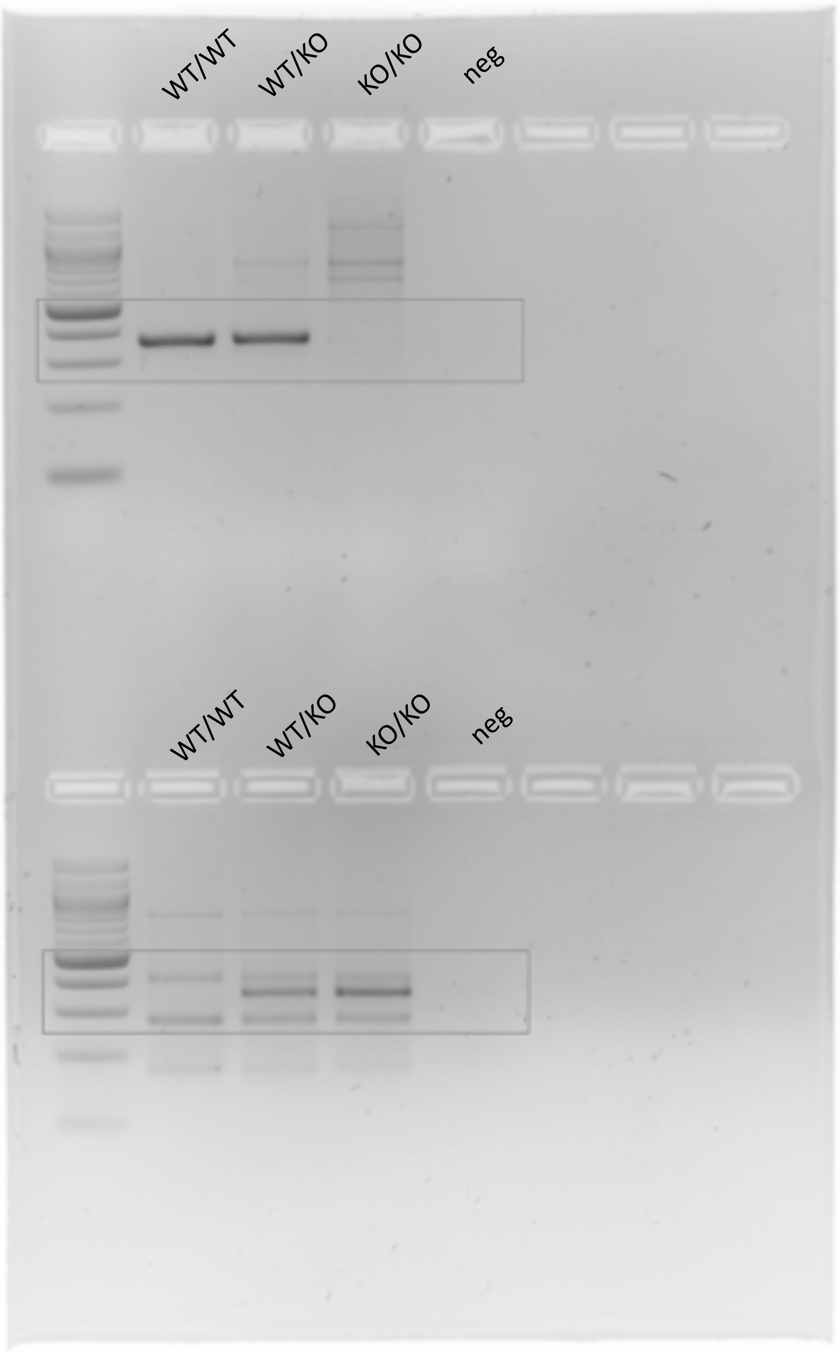

Figure S1c

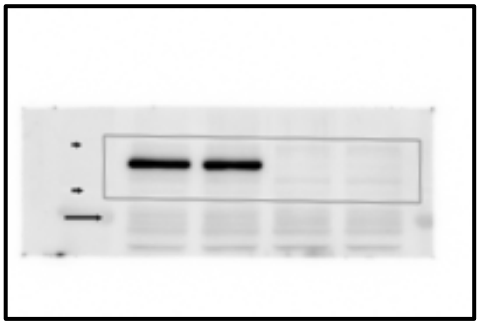

PACS1
(703; ref. 69)

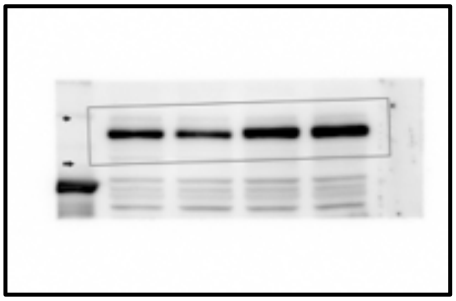

PACS2
(193; ref. 59)

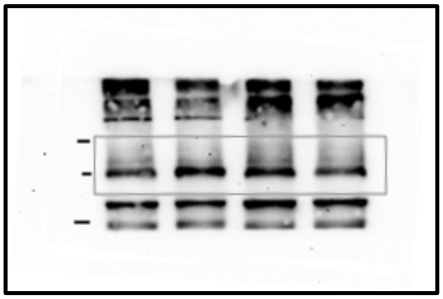

HDAC6
Assay Biotech #C0226

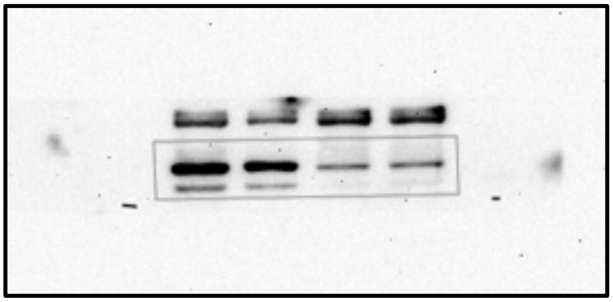

WDR37
Sigma #HPA037565

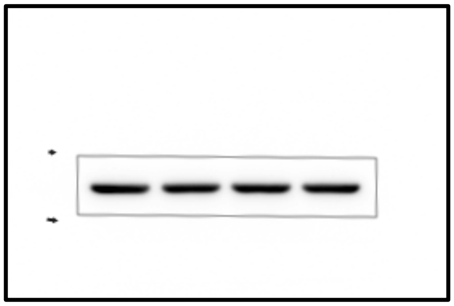

Actin
Millipore #MAB1501

Figure S1d

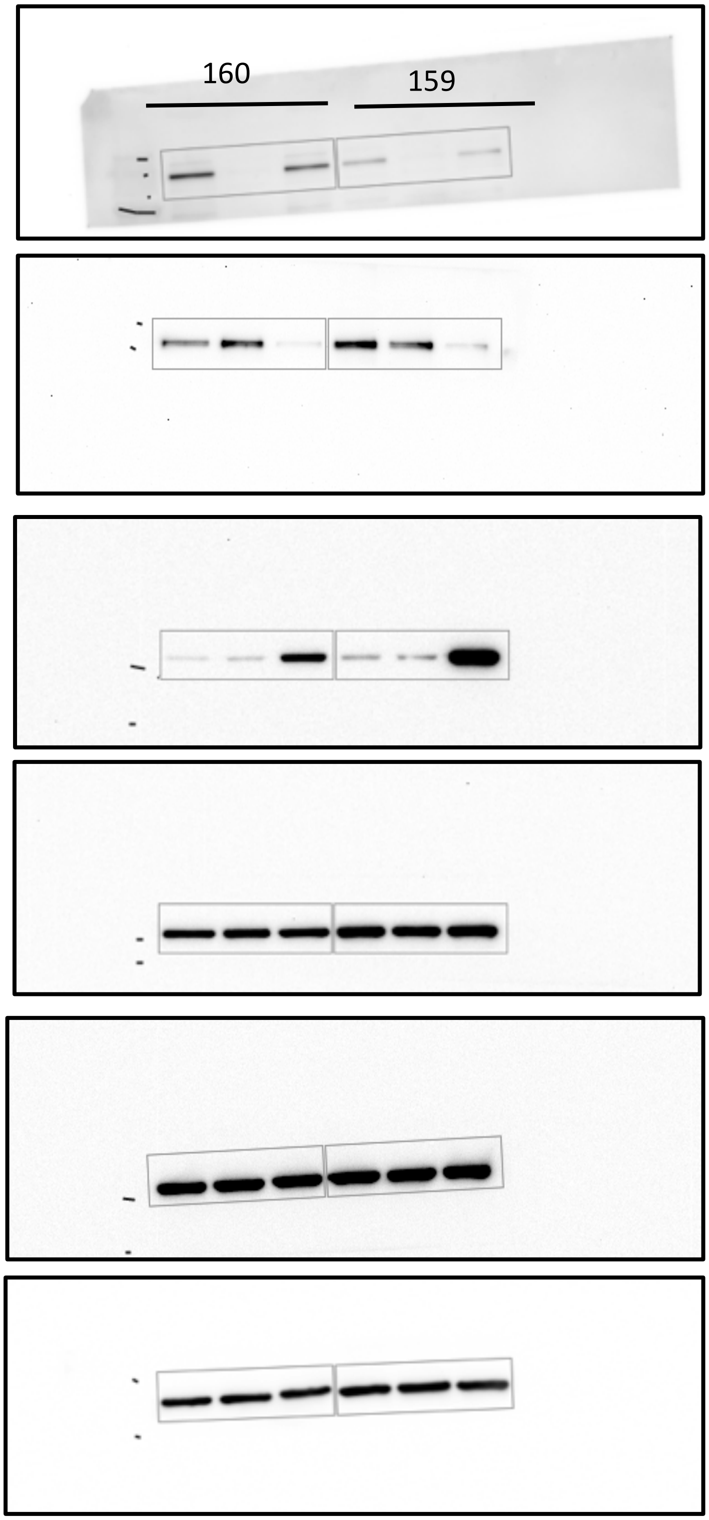

PACS1
(703; ref. 69)

HDAC6
CST #7558S

Ac-α-tubulin
CST #5335S

α-actinin
CST #3134S

α-tubulin
CST #3873S

Actin
Millipore #MAB1501

Figure S2b

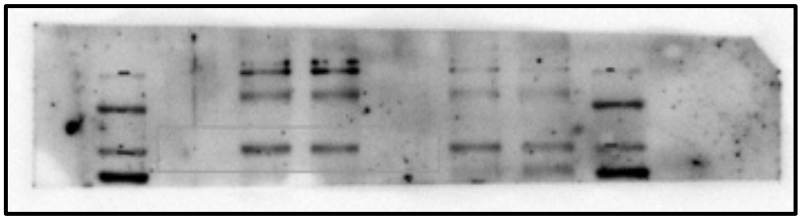

PACS1 coIP
(703; ref. 69)

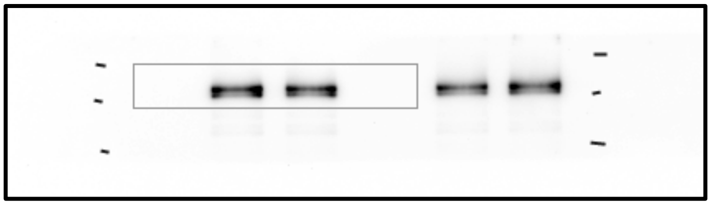

HDAC6 IP
CST #7558S

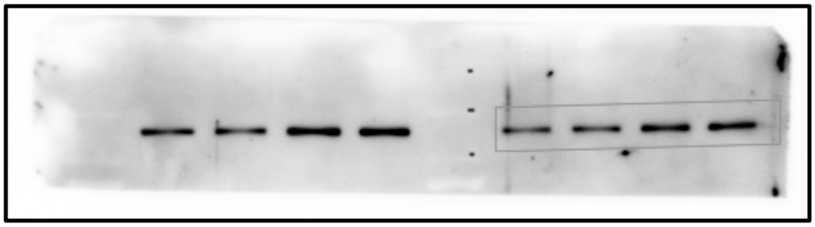

PACS1 input
(703; ref. 69)

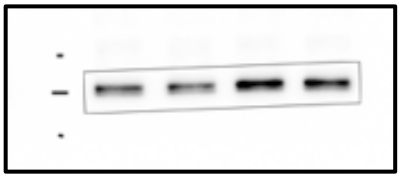

HDAC6 input
CST #7558S

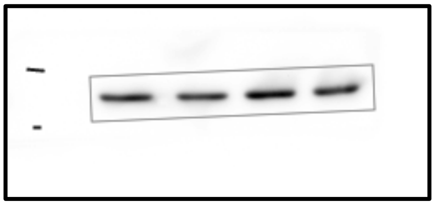

Actin
Millipore #MAB1501

Figure S2c

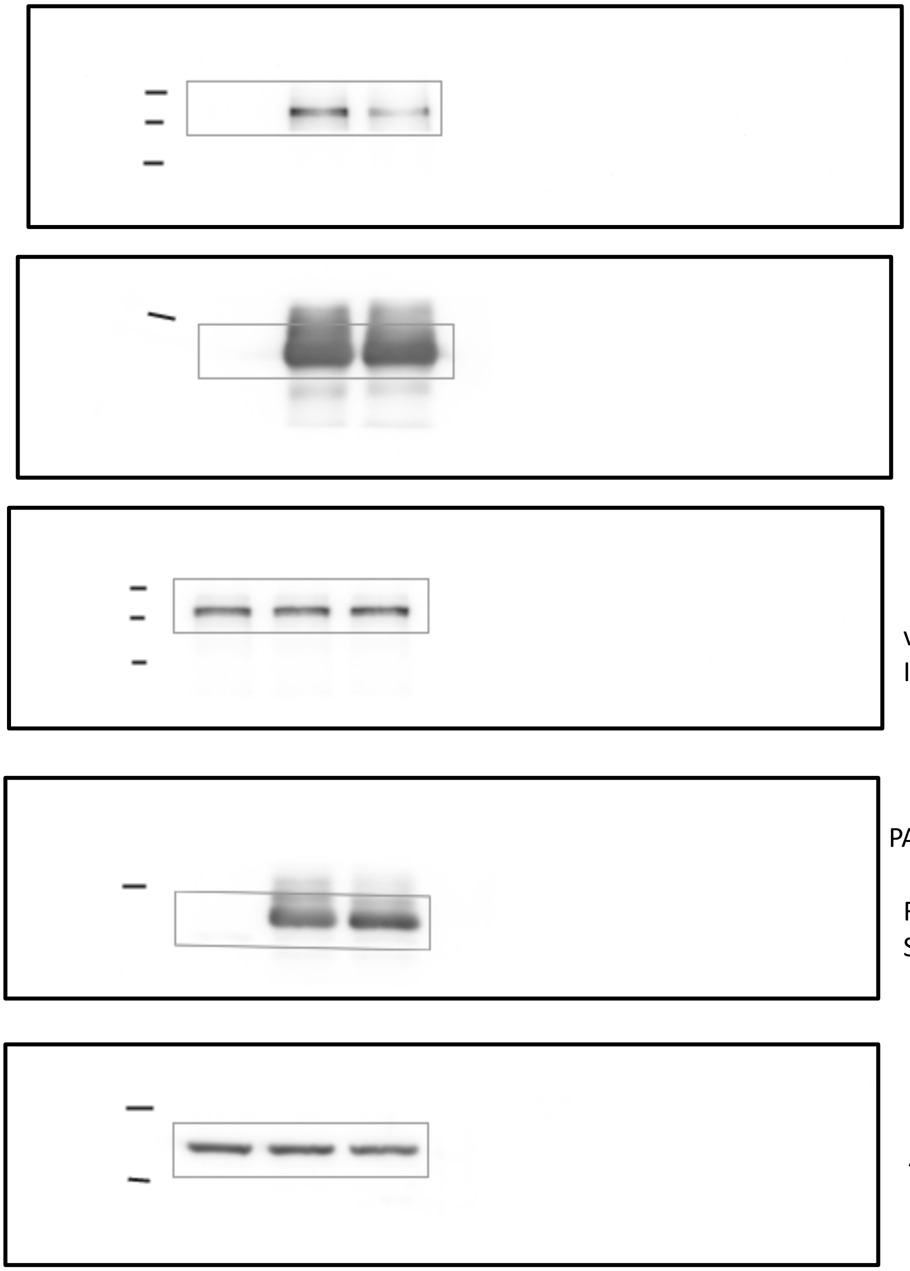

HDAC6-v5 coIP

v5 antibody
Invitrogen #R960-25

PACS1-flag IP

Flag antibody
Sigma F7425

HDAC6-v5 input

v5 antibody
Invitrogen #R960-25

PACS1-flag input

Flag antibody
Sigma F7425

Actin
Millipore #MAB1501

Figure S2d

PCR: NeoF-NeoR primers

PCR: GFPF-GFPR primers

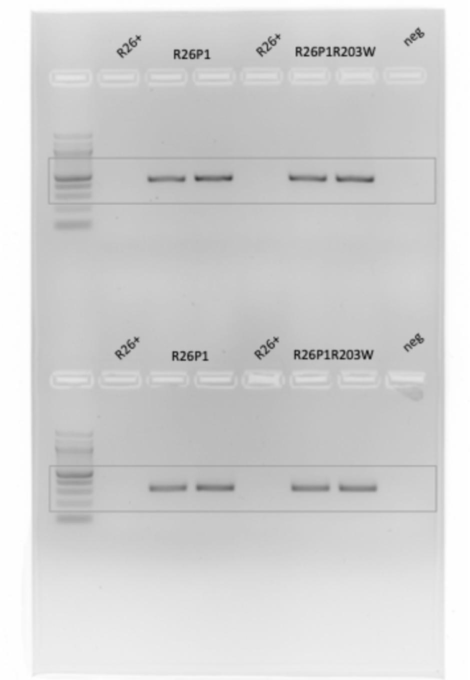

PCR: R26F-SAR-R26R primers

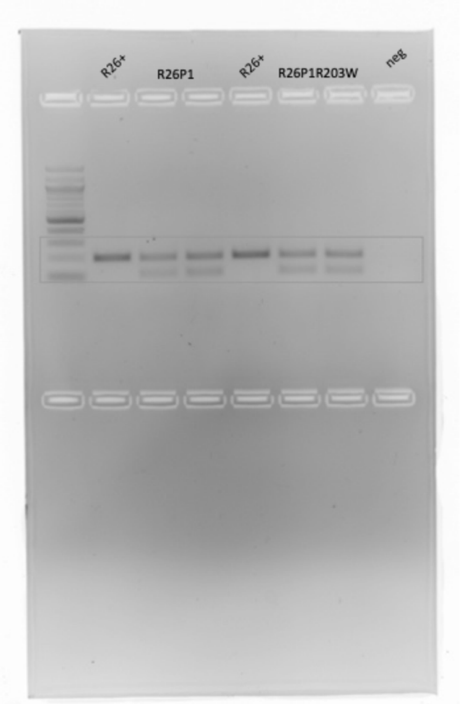

Figure S3a

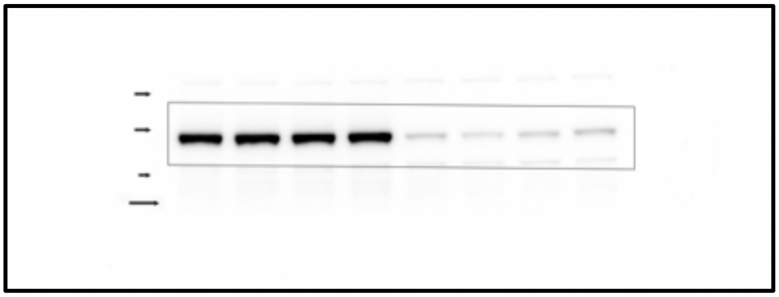

HDAC6
Assay Biotech #C0226

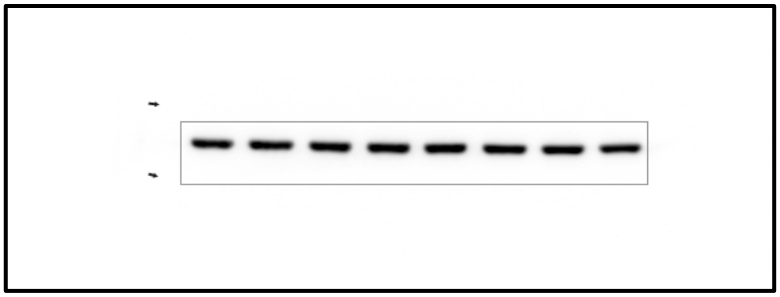

Actin
Millipore #MAB1501

Figure S4a

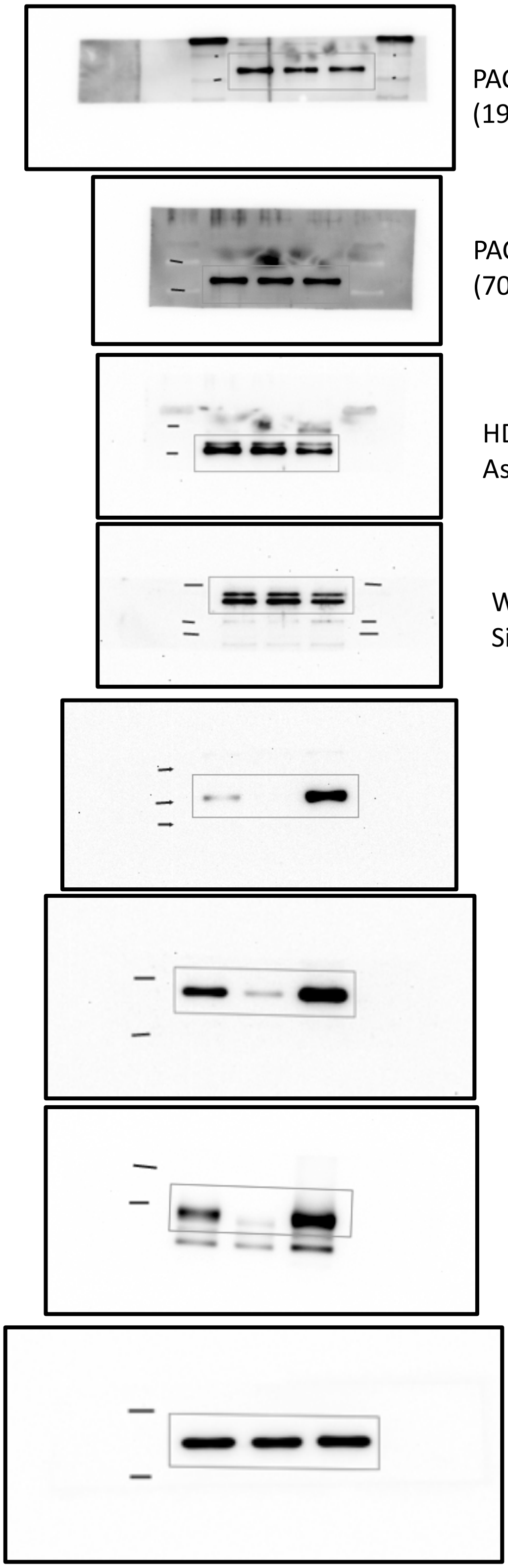

PACS2
(193; ref. 59)

PACS1
(703; ref. 69)

HDAC6
Assay Biotech #C0226

WDR37
Sigma #HPA037565

AMPAR1
CST #13185

GABAaRα1
NeuroMab #75-136-020

PSD95
NeuroMab #75-028-0

Actin
Millipore #MAB1501

Figure S5a

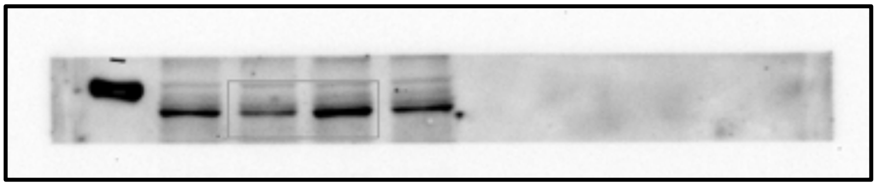

Ac-cortactin
Sigma #09-881

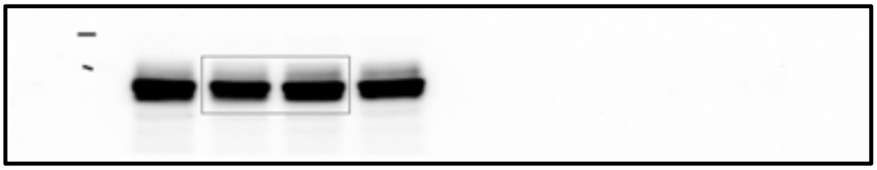

Cortactin
Sigma #05-180

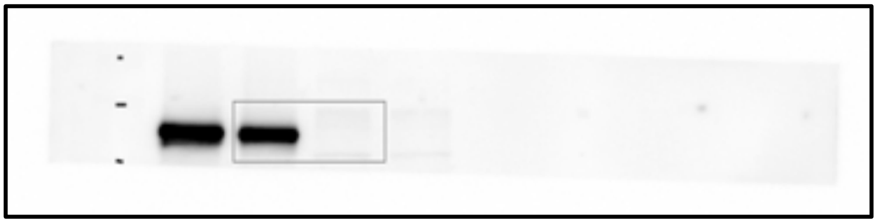

PACS1
(703; ref. 69)

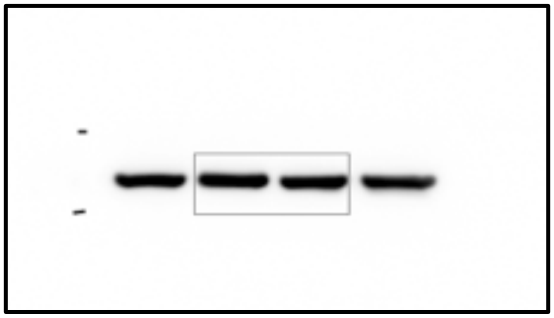

Actin
Millipore #MAB1501

Figure S6b

PCR: i3F-e4R primers

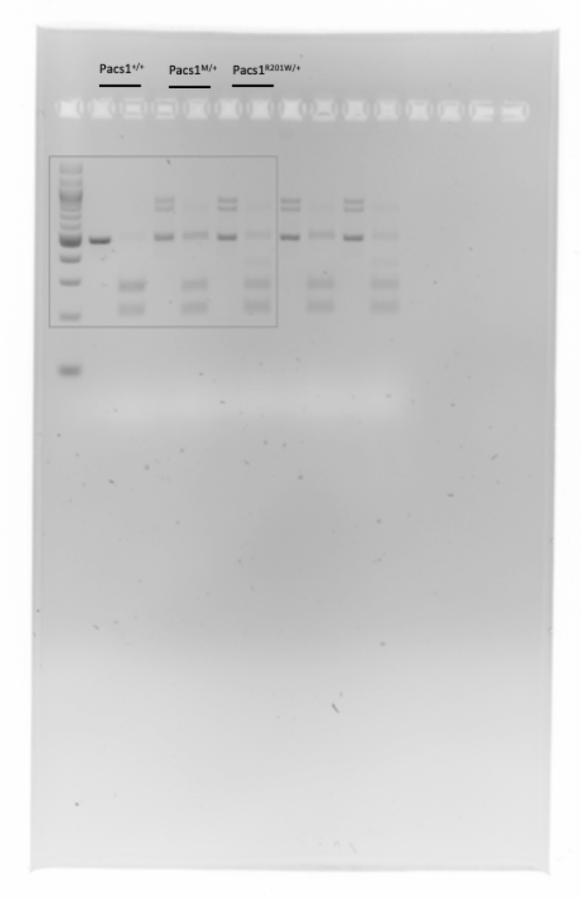

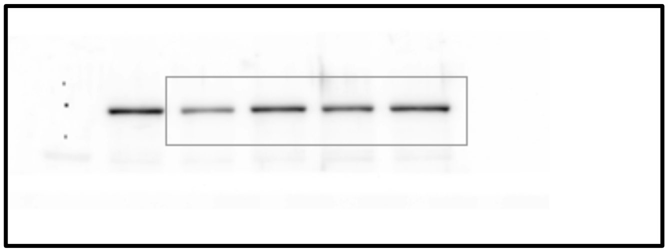

PACS1
(703; ref. 69)

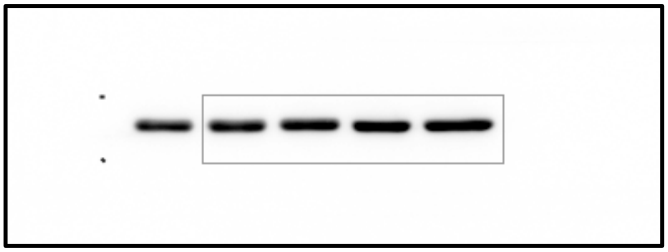

Actin
Millipore #MAB1501

Figure S7a

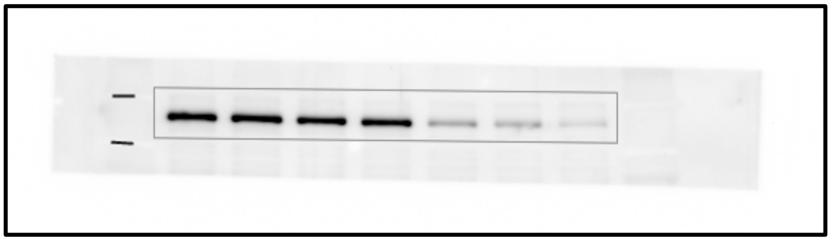

PACS1
(703; ref. 69)

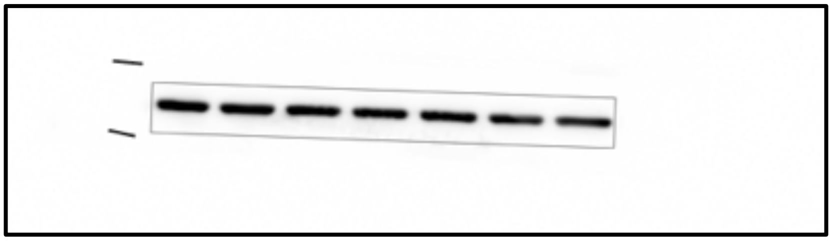

Actin
Millipore #MAB1501

Figure S7c
