# Supplementary material for: Neural deficits in a mouse model of PACS1 syndrome are corrected with PACS1- or HDAC6-targeting therapy
Source: Nat Commun. 2023 Oct 17;14:6547. doi: 10.1038/s41467-023-42176-8 (PMC10582149; doi:10.1038/s41467-023-42176-8)
Supplement: Supplementary file 3 — Description of Additional Supplementary Files [file 41467_2023_42176_MOESM3_ESM.pdf]

Description of Additional Supplementary Files

File Name: Supplementary Movie 1
Description: PACS1R203W causes the Golgi to disseminate into the neurites of hippocampal neurons
