# Supplementary material for: Neural deficits in a mouse model of PACS1 syndrome are corrected with PACS1- or HDAC6-targeting therapy
Source: Nat Commun. 2023 Oct 17;14:6547. doi: 10.1038/s41467-023-42176-8 (PMC10582149; doi:10.1038/s41467-023-42176-8)
Supplement: Supplementary file 5 — Reporting Summary [file 41467_2023_42176_MOESM5_ESM.pdf]

nature portfolio

Corresponding author(s): Gary Thomas

Last updated by author(s): Sep 25, 2023

Reporting Summary

Nature Portfolio wishes to improve the reproducibility of the work that we publish. This form provides structure for consistency and transparency in reporting. For further information on Nature Portfolio policies, see our Editorial Policies and the Editorial Policy Checklist.

Statistics

For all statistical analyses, confirm that the following items are present in the figure legend, table legend, main text, or Methods section.

n/a	Confirmed	
☐	☒	The exact sample size (*n*) for each experimental group/condition, given as a discrete number and unit of measurement
☐	☒	A statement on whether measurements were taken from distinct samples or whether the same sample was measured repeatedly
☐	☒	The statistical test(s) used AND whether they are one- or two-sided<br>*Only common tests should be described solely by name; describe more complex techniques in the Methods section.*
☒	☐	A description of all covariates tested
☐	☒	A description of any assumptions or corrections, such as tests of normality and adjustment for multiple comparisons
☐	☒	A full description of the statistical parameters including central tendency (e.g. means) or other basic estimates (e.g. regression coefficient) AND variation (e.g. standard deviation) or associated estimates of uncertainty (e.g. confidence intervals)
☐	☒	For null hypothesis testing, the test statistic (e.g. F, t, r) with confidence intervals, effect sizes, degrees of freedom and P value noted<br>*Give P values as exact values whenever suitable.*
☒	☐	For Bayesian analysis, information on the choice of priors and Markov chain Monte Carlo settings
☒	☐	For hierarchical and complex designs, identification of the appropriate level for tests and full reporting of outcomes
☐	☒	Estimates of effect sizes (e.g. Cohen's *d*, Pearson's *r*), indicating how they were calculated

Our web collection on statistics for biologists contains articles on many of the points above.

Software and code

Policy information about availability of computer code

Data collection	Nikon NIS-Elements was used for confocal imaging acquisition. Western blot images were captured using a FluorChem E image acquisition system (ProteinSimple). HDAC6 activity assays were measured using a BioTek Synergy 4 microplate reader. Patch-clamp recordings were acquired using Clampex 10.4 (pCLAMP 10.4 software suite, Molecular Devices). qPCR reactions were performed and data acquired using a QuantStudio 3 Real-Time PCR System (Applied Biosystems, A28567).
Data analysis	Statistical analysis were performed in GraphPad Prism 9. Nikon NIS-Elements, Fiji/Image J, Neurolucida 360, and NeuroExplorer 360 were used for the analysis of confocal images. Deconvolution was performed with Huygens Professional v22.10 (Scientific Volume Imaging). Golgi topology was performed with Imaris 10.0.0 (Bitplane). Densitometry quantification of Western Blots was performed in AlphaView software (ProteinSimple). Patch-clamp recordings were analyzed using Clampfit (pCLAMP 10.4 software suite, Molecular Devices). Real-time PCRs were quantified using Applied Biosystems Design and Analysis Software v2.6.0.

For manuscripts utilizing custom algorithms or software that are central to the research but not yet described in published literature, software must be made available to editors and reviewers. We strongly encourage code deposition in a community repository (e.g. GitHub). See the Nature Portfolio guidelines for submitting code & software for further information.

Data

Policy information about availability of data

All manuscripts must include a data availability statement. This statement should provide the following information, where applicable:

- Accession codes, unique identifiers, or web links for publicly available datasets
- A description of any restrictions on data availability
- For clinical datasets or third party data, please ensure that the statement adheres to our policy

All data generated or analyzed during this study are included in the published article and its supplementary file. Source data are provided with this paper.

Human research participants

Policy information about studies involving human research participants and Sex and Gender in Research.

Reporting on sex and gender	N/A
Population characteristics	N/A
Recruitment	N/A
Ethics oversight	N/A

Note that full information on the approval of the study protocol must also be provided in the manuscript.

Field-specific reporting

Please select the one below that is the best fit for your research. If you are not sure, read the appropriate sections before making your selection.

☒ Life sciences ☐ Behavioural & social sciences ☐ Ecological, evolutionary & environmental sciences

For a reference copy of the document with all sections, see nature.com/documents/nr-reporting-summary-flat.pdf

Life sciences study design

All studies must disclose on these points even when the disclosure is negative.

Sample size	Sample size was determined considering those reported in previous publications that include similar studies and experimental designs (Ageta-Ishihara et al. 2013 (reference 40), Bridi et al. 2020 (reference 51), and Xing et al. 2020 (reference 49)), and based on power analyses using preliminary estimates of variance with the goal of achieving 80% power to observe differences at α=0.05.
Data exclusions	A rigid exclusion criteria were pre-established and strictly respected throughout data collection and analysis.
Replication	All experimental data presented in this study were reproducible in multiple cohorts and experimental replicates and the numbers are indicated in the figure legends.
Randomization	Mice were included in the study based on their genotypes (confirmed presence of control or mutant transgenic PACS1 alleles). Male and female littermates were randomly assigned to different experimental groups. At least 3 litters per genotype were included in the randomization. Patient-derived cells included in this study represented the entire inventory commercially available at the time. Patient-derived cells lines were sequenced upon arrival to confirm the presence of the PACS1 Syndrome mutation (R203W). For cell culture experiments, no randomization was required since control and treated groups derived from the same source cell line.
Blinding	The spine analysis (Figure 5a) was blinded regarding the identity of each experimental group since the identification of secondary dendrites suitable for the analysis depends on the investigator's criteria. Other data was performed non-blinded as data were acquired using automated or semi-automated methods independent of human judgement.

Reporting for specific materials, systems and methods

We require information from authors about some types of materials, experimental systems and methods used in many studies. Here, indicate whether each material, system or method listed is relevant to your study. If you are not sure if a list item applies to your research, read the appropriate section before selecting a response.

Materials & experimental systems

n/a	Involved in the study
☐	☒ Antibodies
☐	☒ Eukaryotic cell lines
☒	☐ Palaeontology and archaeology
☐	☒ Animals and other organisms
☒	☐ Clinical data
☒	☐ Dual use research of concern

Methods

n/a	Involved in the study
☒	☐ ChIP-seq
☒	☐ Flow cytometry
☒	☐ MRI-based neuroimaging

Antibodies

Antibodies used

Antibodies- actin (Millipore, MAB1501, 1:3000), α-actinin (Cell Signaling Technology (CST) 3134S, 1:1000), α-tubulin (DMA1 Cell Signaling 3873S 1:1000 and Thermo Fisher 66031, 1:250), Ac-Lys40-α -tubulin (CST 5335S, 1:1000), cortactin (4F11 Sigma 05-180, 1:1000), Ac-cortactin (Sigma 09-881, 1:1000), CTIP2 (25B6 Abcam 18465, 1:500), SATB2 (Abcam 51502, 1:50), EB1 (BD Biosciences 610534, 1:50), V5 (Invitrogen, R960-25, 1:2000), HDAC6 (Abcam 253033, 1:50, D2E5 CST 7558S 1:1000, and Assay biotech C0226, 1:1000), p62 (Abcam 56416, 1:100), Flag (Sigma-Aldrich, F7425, 1:5000 and A2220, 50% slurry), HA (CST 3724S, 1:4000 and Biolegend 901513, 1:1000), furin (MON-152, kindly provided by J. Creemers, Leuven, 1:100), GAPDH (14C10, CST 2118S, 1:1000), Giantin (kindly provided by Dr. A. Linstedt, CMU, 1:750), GM130 (BD Biosciences 610534, 1:500), pericentrin (AbCam 4888, 1:500), MAP2 (Biolegend 801810, 1:5000), βIII-tubulin (Biolegend 801213, 1:500), Nestin (AbClonal A11861, 1:100), Pax6 (CST 60433, 1:200), Sox2 (D6D9, CST 3579, 1:400), PSD95 (NeuroMab 75-028-020, 1:250), GABAARα1 (NeuroMab 75-136-020, 1:1000), AMPAR1 (CST 13185, 1:1000), WDR37 (Sigma HPA037565, 1:1000), RFP (Rockland 600-401-379, 1:800), PACS1 (BD Biosciences 611371, 1:100, Invitrogen PA558589, 1:100 and Ab 703 69, 1:1000), PACS2 (Ab 193 59, 1:1000), Goat anti-Rabbit IgG Alexa Fluor 488 (Invitrogen A11008, 1:400), Goat anti-Mouse IgG1 Alexa Fluor 568 (Invitrogen A11004, 1:400) Goat anti-Mouse IgG Alexa Fluor 647 (Invitrogen A-21242, 1:400), Goat anti-Chicken IgY Alexa Fluor 633 (Invitrogen A-21103, 1:400), Goat anti-Rat IgG Alexa Fluor 488 (Invitrogen A-11006, 1:400), Goat anti-Rabbit IgG Alexa Fluor 594 (Invitrogen A-11012, 1:400).

Validation

All antibodies are validated by commercial sources or as indicated here. Anti-Giantin (PMID: 7691276); anti-PACS1 (703), reference 69; anti-PACS2 (193) reference 59; anti-human furin (MON-152), PMID:1737642.

Eukaryotic cell lines

Policy information about cell lines and Sex and Gender in Research

Cell line source(s)

HCT116 and Hela cells (Atkins et al., 2014 PMID: 25159152). PACS1 syndrome dermal fibroblasts GM27159 (R203W patient), GM27160 (parent), GM27650 (R203W patient) and GM27651 (parent) were from Coriell Institute and immortalized by transduction with hTERT (LVP1130-Puro, Gentarget). PACS1 syndrome iPSC PACS1002i-GM27159 (R203W patient) and iPSC PACS1001i-GM27160 (parent) were from WiCell. Hela+/P1R203W CRISPR-Cas9 knock-in cells and their WT isogenic control were from System Biosciences (CS721B-1). Pacs1WT and Pacs1Δ4bp/Δ4bp embryonic fibroblasts were isolated from E13.5 littermate embryos and immortalized with a retrovirus expressing SV40 large T antigen (kindly provided by M. Suda, UPMC).

Authentication

HCT116 cells were obtained from B. Vogelstein and validated by multiplex PCR (AMFlSTR®), U2OS cells were from ATCC, and the CRISPR/Cas9 Hela PACS1R203W and isogenic WT control lines were from System Biosciences and sequenced (CS721B-1).

Mycoplasma contamination

The cells are mycoplasma negative.

Commonly misidentified lines
(See ICLAC register)

Hela cells were used in this study and were validated as described above.

Animals and other research organisms

Policy information about studies involving animals; ARRIVE guidelines recommended for reporting animal research, and Sex and Gender in Research

Laboratory animals

Veterinary care provided at the University of Pittsburgh complies with the Public Health Service Policy on Humane Care and Use of Laboratory Animals. Mice are housed in an ABSL1 (Animal Biosecurity Level 1) barrier facility. Any animals entering the facility do so through approved vendors or with enhanced monitoring and quarantine. Biosecurity for the space is additionally maintained through the use of microisolator techniques. The colony is maintained at 20°C and 30-70% humidity. Male and female mice were used in this study in line with AVMA and ARRIVE guidelines. P0 mice were used for preparation of dissociated neurons, P1 mice were used for ASO injections, P18 mice were used for IHC and P25-P35 mice were used for electrophysiology studies. C57BL/6J mice (#:000664, RRID:IMSR_JAX:000664) and B6.129S2-Emx1tm1(cre)Krj/J mice (#005628, RRID:IMSR_JAX:005628) were from Jackson Labs. Pacs2KO mice were described (Aslan et al., 2009 PMID: 19481529). All other engineered mice were generated by the Innovative Technologies Development and Mouse Embryo Services Cores in the Department of Immunology of the University of Pittsburgh, using CRISPR/Cas9 technology directly in C57BL/6J zygotes. All single guide RNAs were produced as described 40. R26P1 (B6;Gt(ROSA)26Sortm1(CAG-LSL-Pacs1)Gath/J, ROSA26-LSL-PACS1-WT), and R26P1R203W (B6;Gt(ROSA)26Sortm1(CAG-LSL-Pacs1-R203W)Gath/J, ROSA26-LSL-PACS1-R203W) lines were generated as described 12; the HA-tagged cDNA for WT human PACS1 or PACS1R203W were cloned into the pR26-GFP-Dest (a gift from Ralf Kuehn (Addgene plasmid # 74283)). Pronuclei of fertilized embryos (C57BL/6J, The Jackson Laboratory), produced by natural mating, were microinjected with a mixture of 0.3 µM EnGen Cas9 protein (NEB M0646T), Rosa26-1 sgRNA (21 ng/µl ≈ 0.66 µM) and either the WT or the R203W targeting vector plasmid (10 ng/µl).

	The Pacs1Δ4bp/+ allele (Pacs1em1Gath, Pacs1-4bp-del, Pacs1-KO) was generated by injecting C57BL/6J zygotes with EnGen Cas9 protein and the guide Pacs1-e4-17Rev target sequence (5'-TACAAGAACCGAACTATCTTGGG-3', chr19:5,160,008-5,160,030, mouse GRCm38/mm10 assembly), which resulted in a 4 bp deletion leading to a frameshift null allele. Pacs1R201W/+ (Pacs1tm1Gath, Pacs1-LSL-R201W) lines was generated using the Easi-CRISPR strategy 41. Briefly, the zygotes were injected with 0.33 µM EnGen Cas9 protein, 21.23 ng/µl (~0.66 µM) sgRNA (Pacs1-i3-18rev, 5'-TAGCTGGACCTGAACCACCAAGG-3' , chr19:5,160,141-5,160,163, mouse GRCm38/mm10 assembly) and 10 ng/µl Pacs1-R201W-cKO Megamer (IDT). Pacs1M/+ (Pacs1tm2Gath, Pacs1-LSL-R201) was identified in the process; the Lox-Stop-Lox cassette was inserted in the intron, but R201 remained unchanged.
Wild animals	Wild animals were not involved in the present study.
Reporting on sex	The analysis of sex-dependent differences was not pursued in this study. Data from both sexes were grouped and equally represented in each experimental group.
Field-collected samples	The present study did not involve field-collected specimens.
Ethics oversight	All animal protocols were approved by the University of Pittsburgh Institutional Animal Care and Use Committee according to NIH guidelines and regulations.

Note that full information on the approval of the study protocol must also be provided in the manuscript.
